# A diagnostic support tool for lumbar spinal stenosis: a self-administered, self-reported history questionnaire

**DOI:** 10.1186/1471-2474-8-102

**Published:** 2007-10-30

**Authors:** Shin-ichi Konno, Shin-ichi Kikuchi, Yasuhisa Tanaka, Ken Yamazaki, You-ichi Shimada, Hiroshi Takei, Toru Yokoyama, Masahiro Okada, Shou-ichi Kokubun

**Affiliations:** 1 Department of Orthopaedic Surgery, Fukushima Medical University School of Medicine, Fukushima City, Fukushima, Japan; 2 Department of Orthopaedic Surgery, Tohoku University, Sendai City, Miyagi, Japan; 3 Department of Orthopaedic Surgery, Iwate Medical College, Morioka City, Iwate, Japan; 4 Department of Orthopaedic Surgery, Akita University, Akita City, Akita, Japan; 5 Department of Orthopaedic Surgery, Yamagata University, Yamagata City, Yamagata, Japan; 6 Department of Orthopaedic Surgery, Hirosaki University, Hirosaki City, Aomori, Japan

## Abstract

**Background:**

There is no validated gold-standard diagnostic support tool for LSS, and therefore an accurate diagnosis depends on clinical assessment. Assessment of the diagnostic value of the history of the patient requires an evaluation of the differences and overlap of symptoms of the radicular and cauda equina types; however, no tool is available for evaluation of the LSS category. We attempted to develop a self-administered, self-reported history questionnaire as a diagnostic support tool for LSS using a clinical epidemiological approach. The aim of the present study was to use this tool to assess the diagnostic value of the history of the patient for categorization of LSS.

**Methods:**

The initial derivation study included 137 patients with LSS and 97 with lumbar disc herniation who successfully recovered following surgical treatment. The LSS patients were categorized into radicular and cauda equina types based on history, physical examinations, and MRI. Predictive factors for overlapping symptoms between the two types and for cauda equina symptoms in LSS were derived by univariate analysis. A self-administered, self-reported history questionnaire (SSHQ) was developed based on these findings. A prospective derivation study was then performed in a series of 115 patients with LSS who completed the SSHQ before surgery. All these patients recovered following surgical treatment. The sensitivity of the SSHQ was calculated and clinical prediction rules for LSS were developed. A validation study was subsequently performed on 250 outpatients who complained of lower back pain with or without leg symptoms. The sensitivity and specificity of the SSHQ were calculated, and the test-retest reliability over two weeks was investigated in 217 patients whose symptoms remained unchanged.

**Results:**

The key predictive factors for overlapping symptoms between the two categories of LSS were age > 50, lower-extremity pain or numbness, increased pain when walking, increased pain when standing, and relief of symptoms on bending forward (odds ratio ≥ 2, p < 0.05). The key predictive factors for cauda equina type symptoms were numbness around the buttocks, walking almost causes urination, a burning sensation around the buttocks, numbness in the soles of both feet, numbness in both legs, and numbness without pain (odds ratio ≥ 2, p < 0.05). The sensitivity and specificity of the SSHQ were 84% and 78%, respectively, in the validation data set. The area under the receiver operating characteristic curve was 0.797 in the derivation set and 0.782 in the validation data set. In the test-retest analysis, the intraclass correlation coefficient for the first and second tests was 85%.

**Conclusion:**

A new self-administered, self-reported history questionnaire was developed successfully as a diagnostic support tool for LSS.

## Background

Lumbar spinal stenosis (LSS) is a well-recognized spinal disorder and a term used to describe a complex set of symptoms, physical findings, and radiological abnormalities caused by a narrowed spinal canal. The presence of a narrow canal in radiographic imaging does not in itself define the syndrome, and a diagnosis of LSS is defined by symptoms and clinical findings that must be supported by radiographic evidence. Computed tomography and magnetic resonance imaging are often non-specific and there may be discrepancies between clinical symptoms and imaging findings in cases of LSS [1-3].

There is no validated gold-standard diagnostic support tool for LSS, and therefore an accurate diagnosis depends on clinical assessment. However, there are few scientific evaluations of the sensitivity and specificity of diagnoses based on clinical history and physical examinations, or appropriate correlations of these data with imaging and operative findings. Katz et al. used the opinion of two expert orthopedic surgeons to define the presence or absence of LSS [4], and found that the factors in the patient history that were most strongly associated with diagnosis of LSS were a higher age, severe lower-extremity pain, and the absence of pain when seated. The physical findings most strongly associated with the diagnosis were a wide-based gait, an abnormal Romberg test, thigh pain following 30 seconds of lumbar extension, and neuromuscular deficits.

There are two categories of leg symptoms caused by LSS [5]. One type of stenosis presents as unilateral radicular pain (the radicular type), with symptoms of pain, burning, numbness and paresthesia following a specific dermatome or dermatomes. The fifth lumbar nerve root associated with L5 stenosis is most commonly involved. The other type of LSS has symptoms with less dermatomal-specific neurogenic claudication, and nerve roots below L5 are most commonly involved. The typical patient presents with complaints of aching, cramping, or a burning sensation in the bilateral legs. Occasionally, numbness is also apparent and some patients complain of bladder dysfunction and sexual difficulties.

Full-blown cauda equina syndrome only occurs in rare instances, but the above symptoms can occur as a part of cauda equina syndrome [6,7]. Therefore, we hypothesized that leg symptoms in LSS might be divided into two categories: a radicular type and a cauda equina type. There are significant differences between the symptoms of these types, but there is also significant overlap between the symptoms. Since both the central canal and foraminal dimensions increase in flexion and diminish in extension, patients with both types of LSS experience exacerbation of symptoms with extension and improvement with flexion.

Assessment of the diagnostic value of the history of the patient requires an evaluation of the differences and overlap of symptoms of the radicular and cauda equina types; however, no tool is available for evaluation of the LSS category. Therefore, we attempted to develop a self-administered, self-reported history questionnaire as a diagnostic support tool for LSS using a clinical epidemiological approach. The aim of the present study was to use this tool to assess the diagnostic value of the history of the patient for categorization of LSS.

## Methods

### Derivation study 1

A series of 137 patients with LSS and 97 with lumbar disc herniation who successfully recovered following surgical treatment in our department during 2000 and 2003 were included in this study (Table 1). Patients with cervical myelopathy, diabetic neuropathy, previous surgery, peripheral vascular disease, inflammatory disorders, and degenerative scoliosis (defined as lateral tilting of more than 10 degrees) were excluded. Each patient was evaluated by the study investigators using a standard protocol. Operative and follow-up visit notes were reviewed to determine if stenosis was confirmed intraoperatively and if symptoms improved following surgery. Nerve root compression resulting exclusively from a herniated nucleus pulposus was not considered as a symptom of LSS.

**Table 1:** Demographic data for patients in derivation study 1

	LSS (n = 137)	LDH (n = 97)
Male (%)	46	58
Female (%)	54	42
Mean age (yr)	68	41
Mean duration of symptoms (mo)	21	5
Cauda equina type intermittent claudication	50	-
Radicular type intermittent claudication	87	-
Findings on MRI		
One level stenosis	102	14
Two level stenosis	23	0
Three level stenosis	12	0

Assessment of history included questions on location, frequency and severity of pain, and on symptoms including numbness, tingling, and provocative factors. The physical examination included a gait-loading test to confirm neurogenic intermittent claudication; this test involves assessment of walking capacity and symptoms, and a neurological examination of motor, sensory, and reflex activity [8]. We investigated symptoms during gait loading and neurological findings just after gait loading. Reflexes were graded from 0 (no response) to 4 (clonus) at the Achilles tendon and patellar tendon, and strength was graded from 0 (no movement) to 5 (normal) at the knee flexors and extensors, ankle dorsiflexors and plantar flexors, and extensor hallucis longus. A pinprick sensation was graded as absent, decreased, or normal at the dorsomedial foot, dorsolateral foot, medial calf, and lateral calf. MRI radiographical reports were abstracted from patient records.

The LSS patients were categorized into radicular and cauda equina types based on history, physical examination, and MRI findings. The radicular type was characterized by symptoms of pain, burning, numbness, and paresthesias following a specific dermatome with radiological evidence of the responsible nerve root compression, which was confirmed if intermittent claudication was abolished following single nerve root infiltration. Patients of the cauda equina type presented some bilateral symptoms related to cauda equina compression syndrome with less dermatomal-specific neurogenic claudication and radiological evidence of cauda equina compression. The cauda euqina type spinal stenosis is distinct from the cauda equina syndrome. A full blown cauda equina syndrome occurs in rare instances in the cauda equina type spinal stenosis. Therefore, urgent surgery is not required in the cauda equina type spinal stenosis. Predictive factors for overlap symptoms between the two types of LSS were derived from the data and factors for predicting the cauda equina type were also determined. Based on the results of univariate analysis for predictors of LSS, a self-administered, self-reported history questionnaire (SSHQ) was developed as a diagnostic support tool for LSS.

### Derivation study 2

This study was performed in six university hospitals, ten medical centers, and thirty one hospitals and clinics affiliated with university hospitals or medical centers during January and March in 2004. A series of 115 patients with LSS gave informed consent to participate in the study and answered the SSHQ before surgery. All these patients recovered following surgery. Patients with cervical myelopathy, diabetic neuropathy, previous surgery, inflammatory disorders, and degenerative scoliosis were excluded. All patients were evaluated by study investigators using the same protocol as that in derivation study 1. Operative and follow-up visit notes were reviewed to determine if stenosis was confirmed intraoperatively and if symptoms improved following surgery. Nerve root compression resulting exclusively from a herniated nucleus pulposus was not considered as a part of LSS syndrome. All LSS patients were categorized into radicular or cauda equina types based on history, physical examination, and MRI findings using the same criteria as those in derivation study 1. There were 55 patients with radicular type LSS and 60 patients with the cauda equina type (Table 2). A responsible nerve root was confirmed if intermittent claudication was abolished following single nerve root infiltration. The sensitivity of each question on the SSHQ was calculated and compared between the radicular and cauda equina types. To assess the cut-off point to distinguish between the types, one point was assigned to each question on the SSHQ, and the clinical prediction rule was defined based on the scores.

**Table 2:** Demographic data for patients in derivation study 2

	Radicular type (n = 55)	Cauda equina type (n = 60)
Male (%)	52	42
Female (%)	48	58
Mean age (yr)	68	71
Mean duration of symptoms (mo)	19	32
Findings on MRI		
One level stenosis	43	47
Two level stenosis	12	10
Three level stenosis	0	3

### Validation study

**Figure 1:**
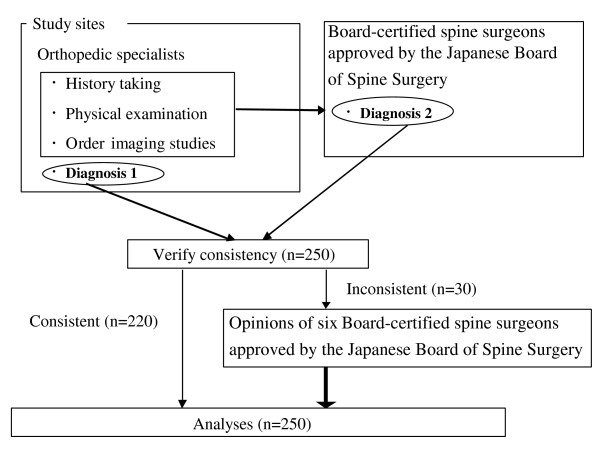
Flow chart of how the diagnosis of LSS was determined.

We prospectively evaluated the association between the diagnosis of LSS and clinical information, including the history and physical examination of patients with leg symptoms. This study was performed in six university hospitals, ten medical centers, and sixty eight hospitals and clinics affiliated with university hospitals or medical centers during July and September in 2004. We enrolled consecutive patients older than 20 years of age with primary symptoms of pain or numbness in the legs. We excluded patients who have been treated by some medical practices within one year before examination. Patients with cervical myelopathy, previous surgery, degenerative scoliosis (defined as lateral tilting of more than 10 degrees) and inflammatory disorders were also excluded. This study included 250 patients who complained of leg symptoms, including cases of LSS (n = 165), lumbar disc herniation (n = 61), diabetic neuropathy (n = 13), and peripheral vascular disease (n = 11) (Table 3). The study was approved by the institutional review board of each study institution as necessary. Written informed consent was obtained from the all patients. The patients gave informed consent and then answered the SSHQ. The following steps were taken to reach a final diagnosis for each of the enrolled patients (Figure 1). In the first step, at each institution the orthopedic physician who saw a patient made the clinical diagnosis based on the history, physical examination, and radiographic findings. In addition, to verify the diagnosis made by each physician, six board-certified spine surgeons approved by the Japanese Board of Spine Surgery also made a diagnosis for each patient based on the clinical information and findings of the MRI. The opinions of six board-certified spine surgeons approved by the Japanese Board of Spine Surgery were used as the gold standard for diagnosis of LSS. The radicular type was characterized by symptoms of pain, burning, numbness, and paresthesias following a specific dermatome with radiological evidence of the responsible nerve root compression, which was confirmed if intermittent claudication was abolished following single nerve root infiltration. Patients of the cauda equina type presented some bilateral symptoms related to cauda equina compression syndrome with less dermatomal-specific neurogenic claudication and radiological evidence of cauda equina compression.

The sensitivity, specificity, likelihood ratio, and area under the receiver operating characteristic (ROC) curve were estimated. 217 patients classified by investigators as suffering from lower back pain without a significant change in symptoms were given the SSHQ two weeks later during an outpatient visit, and the test-retest reliability over two weeks was investigated in these patients.

**Table 3:** Demographic data for patients in the validation study

	LSS (n = 165)	The others (n = 85)
Male (%)	47	51
Female (%)	53	49
Mean age (yr)	71	48
Mean duration of symptoms (mo)	28	24
Clinical impressions of patient condition	Cauda equina type 78	LDH* 61
	Radicular type 87	DN* 13
		PAD* 11
Findings on MRI		
One level stenosis	127	12
Two level stenosis	31	2
Three level stenosis	7	0

* LDH: Lumbar Disc Herniation, DN: Diabetic Neuropathy, PAD: Peripheral Artery Disease

### Statistical analysis

History and physical examination variables were dichotomized at clinically sensible cut-off values. Pinprick, strength, and Achilles reflexes were each classified as always normal or with at least 1 abnormal finding. Univariate analyses were performed to derive predictors of LSS using logistic regression analysis. Two-by-two contingency tables were prepared to calculate the sensitivity, specificity, and likelihood ratio of the SSHQ. The area under the ROC curve for the derivation data set was estimated to investigate the internal validity of the clinical prediction rule, and the area under the ROC curve for the validation data set was estimated to examine the external validity. Reliability was investigated based on the reproducibility in the test-retest method. Test-retest analysis was performed in 217 patients with a 14-day period between the first and second tests. Test-retest data were examined graphically by plotting the difference between tests against the mean of the 2 tests [9]. The intraclass correlation coefficient of the SSHQ score for the first and second tests was calculated to confirm reproducibility. The κ coefficient was calculated to examine conformity for each item, based on the following criteria: 0 to < 0.2, poor; 0.2 to < 0.4, fair; 0.4 to < 0.6, moderate; 0.6 to < 0.8, substantial; and > 0.8, almost perfect [10]. All the studies were approved by the ethics committee of Fukushima Medical University.

## Results

### Univariate analysis for predictors of LSS

Key factors for predicting overlapping symptoms between the two types of LSS are shown in Table 4. Five history findings had an odds ratio ≥ 2 or p < 0.05: age > 50, lower-extremity pain or numbness, increased pain when walking, increased pain when standing, and improvement of symptoms on bending forward. No physical examination finding had an odds ratio ≥ 2 or p < 0.05. There was no significant difference in the odds ratio of all the predictive factors except for age. Key factors for predicting the cauda equina type of LSS are shown in Table 5. Six history findings had an odds ratio ≥ 2 or p < 0.05: numbness around the buttocks, walking nearly causes urination, a burning sensation around the buttocks, numbness in the soles of both feet, numbness in both legs, and numbness without pain. Physical examination findings with an odds ratio ≥ 2 or p < 0.05 included the absence of or a weak Achilles reflex response.

**Table 4:** Univariate analyses for factors from the MD and MRI data sheets associated with a diagnosis of LSS

	LSS (-) (n = 97)	LSS (+) (n = 137)	Odds Ratio	95% CI	p-value
Age (years) > 50	20.6%	94.9%	71.50	28.9 – 176.9	< 0.001
Gender (Female)	42.0%	54.0%	1.60	0.95 – 2.71	07
Symptoms					
Leg pain or numbness (+)	87.6%	94.9%	2.62	0.99 – 6.93	0.045
Low back pain (+)	72.2%	65.0%	0.72	0.41 – 1.26	0.245
Worse when walking but relieved by taking a rest	18.6%	94.2%	70.77	29.39 – 170.4	< 0.001
Numbness in both legs (+)	15.5%	24.8%	1.80	0.92 – 3.54	0.083
Numbness in the soles of both feet (+)	13.4%	20.4%	1.66	0.81 – 3.40	0.163
Numbness around the buttocks (+)	9.3%	15.3%	77	77 – 4.05	173
Numbness without pain	8.2%	11.7%	1.53	0.63 – 3.75	0.344
A burning sensation around the buttocks	6.2%	8.2%	0.94	0.32 – 2.80	0.912
Walking nearly causes urination	3.1%	5.1%	1.69	0.43 – 6.70	0.452
Worse when standing for a while	24.7%	84.7%	11.38	6.20 – 20.91	< 0.001
Symptoms improve on bending forward	8.1%	72.3%	25.47	11.66 – 55.64	< 0.001
Physical Examination					
Straight Leg Raising test positive	33.0%	21.9%	0.57	0.32 – 1.02	0.058
Symptoms induced by having patients bend forward (+)	30.9%	20.4%	0.57	0.32 – 1.04	0.067
Symptoms induced by having patients bend backward (+)	53.6%	62.0%	1.38	0.81 – 2.35	0.229
Abnormal manual muscle strength test 1)	8.2%	10.2%	1.27	0.51 – 3.15	0.611
Sensory disturbance					
(-)	57.7%	49.6%	reference		
(+) 2)	37.1%	45.3%	1.40	0.82 – 2.38	0.214
Missing data	5.2%	5.1%	0.99	0.30 – 3.22	0.988
Achilles tendon reflex					
Normal	51.5%	48.2%	reference		
Abnormal 3)	43.3%	46.7%	1.15	0.68 – 1.94	0.605
Missing data	5.2%	5.1%	0.99	0.30 – 3.22	0.988
Patellar tendon reflex					
Normal	70.1%	62.8%	reference		
Abnormal 3)	24.7%	32.1%	1.44	0.80 – 2.58	0.221
Missing data	5.2%	5.1%	0.99	0.30 – 3.22	0.988

1) MMT < = 3, Strength was graded from 0 (no movement) to 5 (normal) at the knee extensors, ankle dorsiflexors, and plantar flexors, and extensor hallucis longus.

2) Hypoesthesia, analgesia, or hyperalgesia at the medial knee, dorsal foot, plantar foot, and perineal lesion

3) Absence or low response of deep reflexes

**Table 5:** Univariate analyses for factors from the MD and MRI data sheets associated with a diagnosis of the cauda equina type of LSS

	Radicular type (n = 87)	Cauda Equina type (n = 50)	Odds Ratio	95% CI	p-value
Age (years) > 50	94.3%	96.0%	1.46	0.27 – 7.84	0.655
Gender (Female)	51.7%	56.0%	1.20	0.60 – 2.38	0.608
Symptoms					
Leg pain or numbness (+)	97.7%	96.0%	0.56	0.08 – 4.14	0.569
Low back pain (+)	65.5%	62.0%	0.86	0.42 – 1.77	0.679
Worse when walking but relieved by taking a rest	96.6%	96.0%	0.86	0.14 – 5.31	0.868
Numbness in both legs (+)	11.5%	82.0%	35.08	13.20 – 93.19	< 0.001
Numbness in the soles of both feet (+)	6.9%	78.0%	47.86	16.49 – 138.9	< 0.001
Numbness around the buttocks (+)	11.5%	68.0%	6.36	6.74 – 39.73	< 0.001
Numbness without pain	10.3%	37.0%	5.31	2.17 – 13.01	< 0.001
A burning sensation around the buttocks	6.9%	34.0%	6.95	2.52 – 19.19	< 0.001
Walking nearly causes urination	4.6%	26.0%	7.29	2.23 – 23.86	< 0.001
Worse when standing for a while	92.0%	82.0%	0.40	0.14 – 1.15	0.08
Symptoms improve on bending forward	86.2%	74.0%	0.46	0.19 – 1.10	0.07
Physical Examination					
Straight Leg Raising test positive	21.8%	22.0%	1.01	0.44 – 2.34	0.983
Symptoms induced by having patients bend forward (+)	19.5%	22.0%	1.16	0.49 – 2.73	0.731
Symptoms induced by having patients bend backward (+)	63.2%	58.0%	0.80	0.39 – 1.64	0.546
Abnormal manual muscle strength test 1)	9.2%	12.0%	1.35	0.44 – 4.13	0.602
Sensory disturbance					
(-)	49.4%	50.0%	reference		
(+) 2)	43.7%	46.0%	1.40	0.82 – 2.38	0.214
Missing data	6.9%	4.0%	0.56	0.11 – 2.90	0.486
Achilles tendon reflex					
Normal	65.5%	30.0%	reference		
Abnormal 3)	27.6%	66.0%	5.10	2.41 – 10.79	< 0.001
Missing data	6.9%	4.0%	0.56	0.11 – 2.90	0.486
Patellar tendon reflex					
Normal	63.2%	62.0%	reference		
Abnormal 3)	29.9%	34.0%	1.21	0.57 – 2.54	0.617
Missing data	6.9%	4.0%	0.56	0.11 – 2.90	0.486

1) MMT < = 3, Strength was graded from 0 (no movement) to 5 (normal) at the knee extensors, ankle dorsiflexors, and plantar flexors, and extensor hallucis longus.

2) Hypoesthesia, analgesia, or hyperalgesia at the medial knee, dorsal foot, plantar foot, and perineal lesion

3) Absence or low response of deep reflexes

### A self-administered, self-reported history questionnaire (SSHQ)

Based on the results of univariate analysis for predictors of LSS, we developed the SSHQ as a diagnostic support tool for LSS (see Additional File 1 and 2). The SSHQ included the following questions:

Q1: Numbness and/or pain in the thighs down to the calves and shins.

Q2: Numbness and/or pain increase in intensity after walking for a while, but are relieved by taking a rest.

Q3: Standing for a while brings on numbness and/or pain in the thighs down to the calves and shins.

Q4: Numbness and/or pain are reduced by bending forward.

The key questions for diagnosis of cauda equina symptoms were as follows:

Q5: Numbness is present in both legs.

Q6: Numbness is present in the soles of both feet.

Q7: Numbness arises around the buttocks.

Q8: Numbness is present, but pain is absent.

Q9: A burning sensation arises around the buttocks.

Q10: Walking nearly causes urination.

### Clinical prediction rule

**Figure 2:**
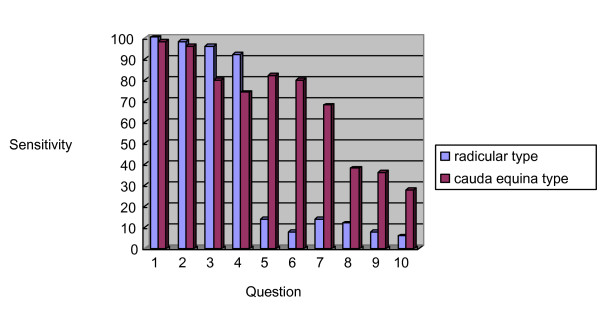
Comparison of the sensitivity of each question for radicular and cauda equina types of LSS.

**Figure 3:**
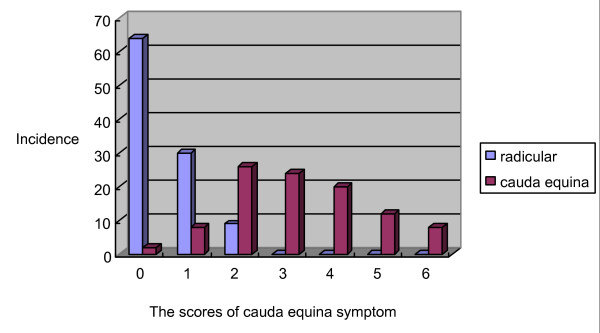
Cut-off point to distinguish between the categories.

The sensitivity of each question in the derivation study was calculated for the radicular and cauda equina types of LSS. The sensitivity differed significantly between the categories (Figure 2). To assess the cut-off point to distinguish between the two types, each question was assigned one point. The scores of predictors of cauda equina symptoms (Q5-Q10) were significantly different between the categories, and the cut-off point was two (Figure 3). Based on these results, a clinical prediction rule was defined based on the total scores: a score of 4 points on Q1–Q4 indicates the presence of LSS; a score of 4 on Q1–Q4 and < 1 on Q5–Q10 indicates the radicular type of LSS; and a score of > 1 on Q1–Q4 and > 2 on Q5–Q10 indicates the cauda equina type of LSS.

### Validity

**Figure 4:**
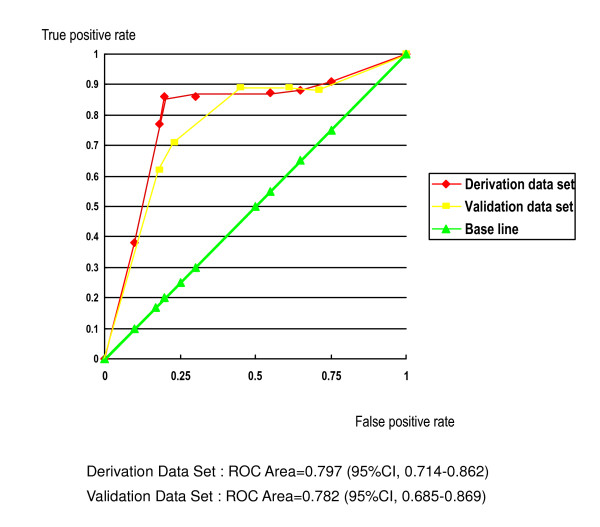
Receiver operating characteristic (ROC) curves for the derivation and validation datasets.

**Figure 5:**
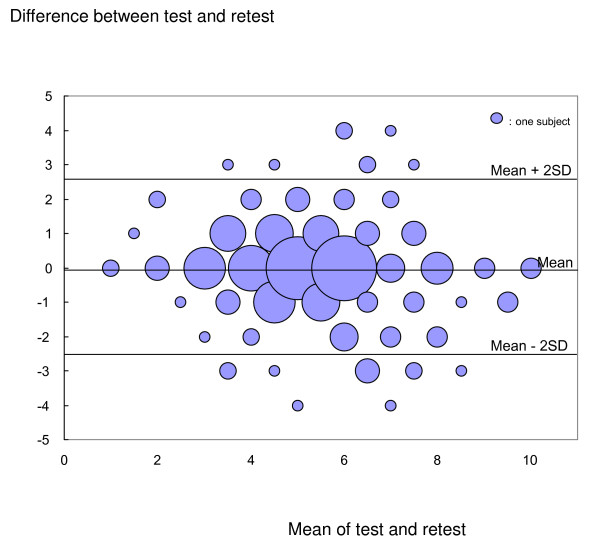
Scatter plot of differences versus the means of the test and the retest.

It took respondents an average of about 1 minute to answer the 10 questions on the SSHQ. Performance indices for the clinical prediction rule are shown in Table 6. The area under the ROC curve was 0.797 in the derivation set and 0.782 in the validation data set (Figure 4). These findings indicate that the SSHQ has both internal and external validity as a diagnostic tool for LSS. The difference between tests plotted against the mean of the tests indicated no obvious relationship or bias (Figure 5). The intraclass correlation coefficient of the SSHQ score for the first and second tests was 0.85, which indicates sufficient reproducibility. One item of the κ coefficient was found to be "fair" (question 8), and all other items were rated as having a conformity of moderate or above.

**Table 6:** Performance indices for the clinical prediction rule

	Estimate	
Index	Derivation Data Set (n = 234)	Validation Data Set (n = 250)
Sensitivity	0.855	0.843
Specificity	0.791	0.781
Likelihood Ratio		
Positive Test Result	1.951	1.886
Negative Test Result	0.201	0.214
Area under the ROC curve	0.797	0.782

Positive criteria: Total score ≥ 4 (Q1–Q4) or Total score ≥ 1 (Q1–Q4) and ≥ 2 (Q5–Q10)

## Discussion

Spinal stenosis patients frequently present with few objective physical findings. Jonsson and Stromqvist found that about 65% of patients have decreased walking ability [11], but up to 95% of patients treated surgically have only subjective symptoms, principally pain [12,13]. Furthermore, diagnostic imaging cannot be used reliably to diagnose LSS, since CT and MRI are often non-specific and do not prove that symptoms arise from nerve root compression [14,15]. A systematic review of original diagnostic studies on LSS revealed that no firm conclusions about the diagnostic performance of the different tests could be drawn due to heterogeneity and overall poor quality[16].

We developed a simple clinical diagnostic support tool to identify patients with LSS[17]. Although further studies are needed to validate this tool in primary care settings, it has a sensitivity of 92.8% and a specificity of 72.0%. By asking patients, who presented with back and leg symptoms suggestive of LSS, to fill out a simple questionnaire, consisting of five questions on their medical history (age and history of diabetes) and symptoms (presence or absence of intermittent claudication, aggravation of symptoms by standing and relief of symptoms by forward bending) followed by a short clinical examination checking the postural changes in their leg symptoms, Achilles' tendon reflex, SLR test and the measurement of ABI, the diagnosis of LSS can be established by high sensitivity and spcificity without obtaining MRI. Therefore, misdiagnosis or underdianosis of LSS at the primary care levels can be minimized and patients have a greater chance to get access to appropriate medical service by a referral to a spine specialist. A self-administered, self-reported history questionnaire as a diagnostic support tool for LSS might be more useful for clinician or patients. Therefore, we attempted to develop a self-administered, self-reported history questionnaire as a diagnostic support tool for LSS using a clinical epidemiological approach. To make an accurate diagnosis of LSS from history findings, we categorized the condition into radicular and cauda equina types. A comparison of the sensitivity of each question on the SSHQ showed both overlap and differences between the two categories (Figure 1). These findings suggest that the category of LSS requires consideration to make an accurate diagnosis. The scoring system includes a cut-off point to distinguish the radicular type from the cauda equina type (Figure 2). About 50% of radicular-type cases show relief of symptoms at six months after nerve root block and a further 17% improve with more time after nerve root block. In the cauda equina type, nerve root block is not efficient in relieving the symptoms and therefore surgical intervention is recommended [18]. Therefore, we consider that it is important to define the type of neurogenic intermittent claudication before selecting the therapeutic method, since surgery may be avoidable in certain cases.

The 10 items on the SSHQ for diagnosis of LSS require answers of either "yes" or "no" to minimize any difficulty with responses. As noted above, it took respondents an average of about 1 minute to answer the questions, which indicates that the questionnaire was easy to understand. However, the study has several limitations. First, there is no gold standard for diagnosis of LSS, but in the absence of valid objective criteria we believe that expert opinion is a reasonable strategy for making a diagnosis of a clinical syndrome, and this approach has been used for a variety of disorders. Therefore, we used LSS diagnoses made by six board-certified spine surgeons in our validation study. Second, we did not use logistic regression and multivariate models. Based on the results of this paper, we are planning to use logistic regression and multivariate models in the next project according to STARD checklist for reporting diagnostic accuracy studies [19]. We also note that a larger prospective derivation and validation studies might reveal additional independent factors that correlate with diagnosis of LSS.

## Conclusion

The newly developed self-administered, self-reported history questionnaire can be used for diagnosis of LSS with high sensitivity, specificity, and reproducibility.

## Competing interests

The author(s) declare that they have no competing interests.

## Authors' contributions

SKonno, SKikuchi, SKokuban, YT, YS, KY, MO, TY and HT conceived the study and participated in the study design. SK performed the statistical analysis. SK drafted the initial manuscript for journal submission and participated in revisions. SKonno, SKikuchi, SKokuban, YT, YS, KY, MO, TY and HT coordinated data collection at each site. All authors read and approved the final manuscript.

## Pre-publication history

The pre-publication history for this paper can be accessed here:



## Supplementary Material

Additional file 1 Japanese version of the SSHQ. A copy of the Japanese version of the SSHQ for Japanese readers Click here for file

Additional file 2 English version of the SSHQ. A copy of the English version of the SSHQ Click here for file
