# Supplementary material for: A diagnostic support tool for lumbar spinal stenosis: a self-administered, self-reported history questionnaire
Source: BMC Musculoskelet Disord. 2007 Oct 30;8:102. doi: 10.1186/1471-2474-8-102 (PMC2176057; doi:10.1186/1471-2474-8-102)
Supplement: Additional file 1 — Japanese version of the SSHQ. A copy of the Japanese version of the SSHQ for Japanese readers [file 1471-2474-8-102-S1.doc]

以下の項目は、腰部脊柱管狭窄を診断するための項目です。項目を読みながら、あなたの症状を考えてみて下さい。あなたの症状にあてはまる場合には『はい』に、あてはまらない場合には『いいえ』に○をつけて下さい。

1. 太ももからふくらはぎやすねにかけて､しびれや痛みがある。 はい いいえ
2. しびれや痛みはしばらく歩くとつよくなり､休むと楽になる。 はい いいえ
3. しばらくたっているだけで太ももからふくらはぎやすねにかけてしびれたり痛くなる。

はい いいえ

1. 前かがみになると､しびれや痛みは楽になる。 はい いいえ
2. しびれはあるが痛みはない。 はい いいえ
3. しびれや痛みは足の両側にある。 はい いいえ
4. 両足の裏側にしびれがある。 はい いいえ
5. おしりのまわりにしびれがでる。 はい いいえ
6. おしりのまわりにほてりがでる。 はい いいえ
7. 歩くと尿が出そうになる。 はい いいえ
