# Supplementary material for: A diagnostic support tool for lumbar spinal stenosis: a self-administered, self-reported history questionnaire
Source: BMC Musculoskelet Disord. 2007 Oct 30;8:102. doi: 10.1186/1471-2474-8-102 (PMC2176057; doi:10.1186/1471-2474-8-102)
Supplement: Additional file 2 — English version of the SSHQ. A copy of the English version of the SSHQ [file 1471-2474-8-102-S2.doc]

This list contains some sentences that people have used to describe themselves when they have lumbar spinal stenosis. As you read the list, think of yourself. When you read a sentence that describes you, please circle “yes”. If the sentence does not describe you, please circle “no”.

1. Numbness and/or pain in the thighs down to the calves and shins. Yes No
2. Numbness and/or pain increase in intensity after walking for a while, but are relieved by taking a rest. Yes No
3. Standing for a while brings on numbness and/or pain in the thighs down to the calves and shins. Yes No
4. Numbness and/or pain are reduced by bending forward. Yes No
5. Numbness is present in both legs. Yes No
6. Numbness is present in the soles of both feet. Yes No
7. Numbness arises around the buttocks. Yes No
8. Numbness is present, but pain is absent. Yes No
9. A burning sensation arises around the buttocks. Yes No
10. Walking nearly causes urination. Yes No
